# Regulating riparian forests for aquatic productivity in the Pacific Northwest, USA: addressing a paradox

**DOI:** 10.1007/s11356-015-5814-7

**Published:** 2015-11-27

**Authors:** Michael Newton, George Ice

**Affiliations:** Department of Forest Engineering, Resources and Management, Oregon State University, Corvallis, OR 97331 USA; George Ice, Forest Hydrologist, 24554 Alpine Road, Monroe, OR 97456 USA

**Keywords:** Pacific Northwest, Riparian forest, Salmonid, Aquatic productivity, Water quality, Water temperature

## Abstract

Forested riparian buffers isolate streams from the influence of harvesting operations that can lead to water temperature increases. Only forest cover between the sun and stream limits stream warming, but that cover also reduces in-stream photosynthesis, aquatic insect production, and fish productivity. Water temperature increases that occur as streams flow through canopy openings decrease rapidly downstream, in as little as 150 m. Limiting management options in riparian forests restricts maintenance and optimization of various buffer contributions to beneficial uses, including forest products, fish, and their food supply. Some riparian disturbance, especially along cold streams, appears to benefit fish productivity. Options for enhancing environmental investments in buffers should include flexibility in application of water quality standards to address the general biological needs of fish and temporary nature of clearing induced warming. Local prescriptions for optimizing riparian buffers and practices that address long-term habitat needs deserve attention. Options and incentives are needed to entice landowners to actively manage for desirable riparian forest conditions.

## Introduction

Several species of salmonid fish native to the Pacific Northwest of the USA have been introduced into cold waters of other nations, including the UK, New Zealand, and Chile among others, leading to remarkable sport fisheries. In US states where these fish are native, regulations requiring treed buffer strips to ensure the cold-water habitats are the subject of extensive debate as to their adequacy and design. In countries where they have been introduced (e.g., the UK), such buffers are managed in various ways to provide an array of light spots to enhance photosynthesis in water, leading to nutrition of the fish (Broadmeadow and Nisbet 2004).

Forested streams in northwestern states of the USA are hosts to several salmonid fisheries. Forests in this region are regulated by forest practice rules to protect or enhance these fisheries, in part by reducing the negative impacts of excessive stream temperatures. States regulate timber harvesting near streams to achieve this (Stednick 2008). Riparian (streamside) forests interact with streams by providing shade to limit direct solar radiation that can heat water. Potential negative effects of forest harvesting operations are controlled by retaining strips of forest cover along stream banks (buffers). We postulate that some of the creative management techniques cited by Broadmeadow and Nisbet (2004) offer possible options where salmon are native.

Direct solar radiation warms streams and provides the photosynthesis and primary production that feeds the aquatic food chain. Food availability for fish depends on sunshine. The ecological history of this region’s forests has been dominated by periodic large fires, hence high variability in stream temperature as well as food supply. The current regulatory process is designed to minimize human-caused temperature increases that are trivial compared with natural variation. Rigid interpretation of regulations designed to minimize fluctuations in water quality and the riparian forest environment represents a regulatory paradox that probably minimizes the fish resource as well as forest values.

The State of Oregon has developed a set of rules and water quality criteria since passage of the Forest Practices Act (1971) that constrains how private landowners can harvest trees near streams. This review compares riparian disturbances from natural events and human activities, focused primarily on lessons from the Pacific Northwest and Oregon. We show that: (a) the region’s fisheries are adapted to disturbance events; (b) current levels of disturbance from controlled logging can actually benefit fish populations; (c) site-specific conditions create opportunities to enhance fish populations through riparian forest management; and (d) long-term maintenance of favorable riparian forests requires active management. We propose that the present condition of streams, streamside forests, and local climate be used to guide riparian rules, and hence management, and be directed toward achieving both fishery and riparian forest values.

## Oregon’s regulatory framework

Forest management in Oregon is regulated by the Oregon Forest Practices Act. Oregon’s Departments of Forestry (ODF) and Environmental Quality (ODEQ) have a regulatory objective to “Evaluate the effectiveness of this Act and its rules in encouraging economically efficient forest practices while protecting forest productivity, water and air quality, and fish and wildlife at a variety of scales and over time…” (Oregon Department of Forestry ODF 2014a). In administrative rules (Oregon Department of Forestry ODF 2014b), desired future conditions for riparian management areas are defined in terms of mature forests with no provision for disturbances and regeneration of those buffers. This is despite the need for substantial disturbance where the dominant tree species is likely to be Douglas-fir (*Pseudotsuga menzieii* Mirb. Franco), a shade-intolerant species that needs near-full sunlight to propagate and grow.

Riparian rules for private forests in Oregon define buffers as riparian management areas of specified width (18–30 m; 60–100 ft) along both sides of fish-bearing streams on all private forest lands after timber harvesting is completed. A major focus of buffer regulations is minimizing solar radiation to streams, thus avoiding adverse water temperature increases. This is an effective tool, but constant shade limits aquatic photosynthesis, the source of much stream productivity (Newton and Cole 2005). Many mountain streams are naturally very cold; low valley streams are naturally warmer. Similar buffers are currently required on both. Not only are extensive buffers along very cold streams costly, but heavily shaded streams may be counterproductive to fish as well as forest.

## The stream environment and challenges for rule makers

Stream regulations have greatly reduced short-term changes in water quality (Stednick 2008; Kibler et al. 2013). The Alsea Watershed Study (AWS) (Brown 1970; Stednick 2008) demonstrated that stream protection is needed, especially from mechanical damage and severe burning in the riparian area. It showed that large increases in water temperature can be avoided by maintaining shade. Despite design weaknesses, the AWS revealed the need for protection of stream banks by some sort of buffers. What it and similar studies throughout the Northwest and North America did not reveal was how diverse riparian conditions might benefit fish, how local riparian environments must define or adjust management strategies, and how maintenance is essential for long-term favorable riparian environments.

Riparian forests have many influences on streams. They vary widely in species composition and stand structure (Pabst and Spies 1999; Villarin et al. 2009). They provide organic matter to streams, including nutritious detritus and decomposition products (e.g., Hawkins et al. 1982; Gregory et al. 1987; Kiffney et al. 2003; Wipfli and Musselwhite 2004) that partially support the aquatic food chain. They provide large wood that creates cover from predators and dams that reduce water velocity while creating pool habitat. One of the major functions of riparian forests is to minimize temperature fluctuations and increases in streams by providing shade (Zwieniecki and Newton 1999; Cole and Newton 2013, 2015; and many others). Solar radiation reaching water is inversely related to vegetative cover, of which trees are the most significant type in mature forests. Direct solar radiation is inversely proportional only to tree cover along the southerly sides of water (Zwieniecki and Newton 1999; Cole and Newton 2013).

Shading of streams has benefits and costs, depending on limitations of aquatic food supply and water temperature influences on fish metabolism (Newton and Cole 2005). The value of sunlight for photosynthesis in water is decreased when temperature is excessive. Permanent, no-touch buffers, proposed by some, would limit management of the valuable forest and its requirement for sunlight to regenerate shade-intolerant conifers.

Natural history of the region and its forests must guide rules. The natural variation in forests, climate, and thermal environment of this large region is critical to the function of buffers. The widespread occurrence of Douglas-fir, a light-demanding species native in much of the Pacific Northwest, testifies to the near-universal fire history (i.e., extensive deforestation) where this species has grown. Anthropogenic disturbances, including fires set by natives and early large-scale regeneration harvests, have occurred in all sizes (Van Wagtendonk 2007), adding to lightning strike initiations. Uncontrolled fires in Oregon alone in the last 200 years have led to very large areas with nearly complete deforestation, followed by even-aged forests after decade-requiring natural reforestation. Often these areas burned repeatedly. In western Oregon alone, famous forest fires include the Biscuit, Tillamook (four times), Yaquina (two times), and Nestucca fires, each denuding 20,000 to 240,000 ha (50,000 to 600,000 acres). These events also denuded many river and stream corridors, often for several decades, affecting generations of fish and waters where they occur, apparently contributing to large fish runs.

Even-aged natural stands of Douglas-fir reveal the near-universal roles of fires across most of the forested terrain of the Pacific Northwest, widely known as the “Douglas-fir Region”. A much larger region sharing a similar history includes northern California, Washington, Idaho, and western Montana wherever shade-intolerant Douglas-fir, western larch (*Larix occidentalis*), ponderosa pine (*Pinus ponderosa*), and lodgepole pine (*Pinus contorta*) occur. The famous salmon fishery of this very large and varied region evolved in a setting dominated by extreme disturbance. Such variability in streams and environments suggests a need for rules compatible with local conditions.

Foresters have long recognized that inappropriate timber harvesting and site preparation practices near streams can have negative impacts on aquatic habitats (e.g., Lieberman and Hoover 1948; Schenck 1955). The AWS data stimulated studies quantifying the interaction of shade from buffers in western Oregon on stream water temperature (Brown 1970). These data led to the first Oregon Forest Practices Act rules requiring buffers on fish-bearing streams.

Today, ongoing research programs are increasing the precision with which we assess how forest buffers influence water temperature. The costs and complexity of research that includes both habitat and fish data for variable-buffer studies are high, but the costs of not adjusting for local conditions and restricting management options that could benefit stream productivity are also high.

## Forest streams, how rules must adapt

### Variable streams; fitting rule to local needs

Riparian forests in Oregon, by rule, are managed with a strong emphasis on maintaining favorable water temperature to the extent possible for cold-water fisheries and growing mature riparian forests for large wood recruitment and other functions. The Pacific Northwest states of the USA experience extremely variable stream environments associated with high precipitation in winter and virtually none in summer. The temperature and precipitation interaction changes with elevation and precipitation zones. Surely, many of these streams will benefit from patches of solar radiation to enhance productivity. This interaction needs attention in the rule-making process.

At low elevations of the Pacific Northwest, extreme precipitation occurs in winter months, with negligible contributions to groundwater for the 5 to 6 months of summer. Stream behavior ranges from cold torrents in winter to cool, low-velocity streams that become warmer as discharge declines in summer. Low elevation sites are warmer, on average, than high elevation sites, and may need more protection from heating in summer than is required in mountain streams. In colder streams at higher elevations, fish habitat might benefit from exposure to sun, perhaps in gaps, in order to promote primary production combined with water temperatures that elevate fish metabolism. It is also notable that specific invertebrates, and probably other organisms as well, require open stream habitats that are potentially threatened by uniform mature-forest riparian buffers (Liley 2005).

Oregon’s well-watered terrain above 600 m (2000 ft) has persistent or transient snow packs and large water-holding capacity in deep volcanic soils. Groundwater is very cold when it enters streams in mountainous terrain, and very cool even at lower elevations. Where headwater streams are very cold, fish are small but present in low numbers (Kaczynski 1993); these streams represent a major fraction of regulated stream/kilometers (miles), and are major sources of water for impoundments and rivers. These cold-water streams have not had the research attention given the lower elevation sites, yet buffers are still required at high cost and negative benefit in many instances. Research targeted on alternative riparian practices would reveal appropriate levels of buffer protection. Buffers with broken canopies, as discussed by Broadmeadow and Nisbet (2004), offer combinations of cold water with elevated primary production. Openings in the riparian forest are suggested where the stream is very cold, and mixtures of deciduous and coniferous cover are suggested where the stream is warm.

Most streams follow similar patterns. As they lose altitude, they gain heat as water passes through layer after layer of microclimatic temperature, leading to increasing need for attention to water temperature. There is no strict guide for determining stream temperature change as microclimate warms in the downstream direction. Stratification of sites and their cover by elevation zones appears to be an important area for research activity within the agencies regulating near-stream uses.

Salmonid streams are highly variable in temperature despite widespread productive forests. Rules prohibiting harvesting are costly. Avoiding confiscatory regulations is the responsibility of the regulators. Rules with no benefit to either landowners or fish are avoidable. In one small area of western Oregon (Newton and Zwieniecki 1996, figure four), mean temperatures of six streams with low discharge per unit of basin area were 3 °C (5 °F) warmer 4 miles from their sources than six streams with high discharge per unit area even though the two groups had the same average temperatures upstream. High- and low-discharge streams had significantly different thermal regimes, yet both require identical buffers, one stream never exceeding 15 °C (63 °F). All were in the same county. All were fish bearing. It is difficult to establish relevant rules for managing stream temperature when the streams vary this much in one small area and key factors are not considered.

### Protecting the fishery from overprotection

It is difficult to minimize water temperature increases without adversely affecting productivity of the stream from photosynthesis. Numerous reports of abundant fish associated with forest clearings reveal light as a critical component of aquatic habitat (Newton and Cole 2005). Water temperature and food inevitably interact. Brett et al. (1982) conducted encyclopedic experiments with several salmonid species over a period of 30 years. They elaborated on the interaction between feeding satiety and response to water temperature. The importance of food supply qualified by temperature must guide plans for protective management within wide bounds. Their observations of the range of survivable temperatures and the interaction of feeding level on tolerance to high temperatures provide very useful guidance on conditions to be avoided. They also identified conditions to which fish can adapt, presumably explaining how they can respond positively to huge, vegetation-denuding events.

Brett et al. (1982) showed that fish growth increases with food availability and that food availability influences the optimum temperature for growth. Optimum temperatures increased as fingerlings grew. As fish feeding satiety increased from 60 to 80 to100%, their maximum weight gain was 1.7, 2.5, and 3.2 g/day, respectively. Maximum growth rates of fingerlings occurred at temperatures of 14.8, 17.0, and 18.5 °C, respectively, for the different feeding levels. At each level of satiety, growth was about 90 % of maximum within a range of about 2.5 °C above and below these optimum levels. These anadromous fish thrive under highly variable conditions. Such data offer useful guides for establishing acceptable water temperature levels in accord with feeding opportunity. Observations in streams open to sun have found more and larger fish even at elevated water temperatures (Greene 1950; Murphy et al. 1981; Hetrick et al. 1998b; Leach et al. 2012). For forest streams with salmonids in various environments before and after harvest, the most extreme water temperatures remain only a few hours each day (Cole and Newton 2013), an environment in which fish can compensate for by feeding as soon as water cools (Brett et al. 1982).

Of greater importance than brief peaks of temperature are the mean ranges of temperatures in complete clear-cuts that extend along streams for 300–400 m (1000–1300 ft), which typically show an increase from uncut reaches of 1.2–2.5 °C (2–4 °F) (Cole and Newton 2013), often remaining well within a favorable range. In one stream, these authors reported a maximum temperature of 21 °C (69.4 °F), but cutthroat trout biomass was twice that of uncut units in the same stream. There is strong evidence that maintaining dense overstories of trees over every fish-bearing stream all the time is not necessary and may not be desirable. Newton and Zwieniecki (1996), Zwieniecki and Newton (1999), and Cole and Newton (2013, 2015) have shown that even fractional cover can maintain stream temperature with little change as long as that cover shades the stream from 9 a.m. to 5 p.m. There are numerous reports of fish productivity that increases or is naturally high in streams in or immediately adjacent to openings in forest cover (Murphy et al. 1981; Hawkins et al. 1982; Sedell and Swanson 1984; Wilzbach et al. 1986; Gregory et al. 1987; Hetrick et al. 1998a; Kiffney et al. 2003; Leach et al. 2012; and many others).

These reports provide evidence that salmonid density and biomass can increase with openings in the riparian forest cover (Mellina and Hinch 2009). Clearly, the role of food supply is an important covariant revealing the importance of solar energy when predicting overall fish health in headwater streams. The focus on maximum nonlethal temperatures during the warmest week of the summer masks the potential benefits of openings (Brett et al. 1982).

There may be other reasons why riparian forest management benefits fish in adjacent streams. In cold oligotrophic systems, water temperature and nutrients can be co-limiting to instream primary production. Reduced ocean-derived nutrients have been suggested as a contributing factor to some salmonid production declines on the West Coast of North America (Wipfli et al. 2003). This has even prompted artificial fertilization efforts in some lakes and streams. Nitrate-nitrogen concentrations in streams often increase following timber harvesting (Gravelle et al. 2009). They observed attenuation of nutrient concentrations downstream greater than simple dilution, suggesting biological uptake and processing of increased nutrients. Timber harvesting also increases adjacent stream discharge by reducing evapotranspiration. In some cases, the riparian forest has disproportionately large influences on flow (Hicks et al. 1991). Increased flow, especially during critical low flow periods, can extend the channel network available for fish and make existing habitat more favorable. The simple increase in light by reducing shade may even make it easier for fish to see prey and improve their feeding efficiency.

### Scope of research to guide rules

There is no single management prescription applicable to all streams. Rule-making that prescribes one management approach can be effective for some sites but not others. This becomes increasingly important when applied to small and cold headwater reaches, the majority of stream miles in Oregon, where few streams exceed tolerable temperatures (Kaczynski 1993; Newton and Zwieniecki 1996) but most have very high timber values. One size does not fit all; “conditioning factors” are essential for adjusting Best Management Practices to adapt to variable forest and stream conditions.

Newton and Zwieniecki (1996)) and Cole and Newton (2013) have shown that temperature peaks reached under unbuffered conditions rapidly return to untreated levels within 150–1000 m downstream. They also observed that water temperature varied naturally in reaches as short as 150 m, sometimes warming and sometimes with amplitude of natural variation greater than the accepted variation allowed for human activities by the Protect Cold Water Standard (PCWS) for Oregon of a 0.3 °C (0.5 °F).

Shade on the water during hours of intense sun prevents most warming by solar radiation. Regulating non-shading trees will not result in achieving cooler water temperature goals. Shade in the Northern Hemisphere is provided only by trees in a southerly direction from exposed water, and most shade is generally from trees immediately adjacent to the stream. These are the trees projecting shadows on the water during hours of high-angle sunshine when shade is important; that is, 9 a.m. to 5 p.m. (Newton and Zwieniecki 1996). Buffers north of the water do not shield against direct solar radiation.

The current interpretation of the PCWS (Groom et al. 2011) has little or no relevance for cool streams. Any elevated water temperatures from openings rapidly decline downstream as streams equilibrate with their environment, returning to their natural downstream warming trends. Small, infrequent, and brief water temperature exceedances do not define the overall quality of habitat for fish, especially when food supply is considered (Ice et al. 2007; Loehle et al. 2014).

### Obtaining relevant data for stream temperature and fish

Research is needed to refine relevant site-specific prescriptions. This research must inform regulators about the consequences of management choices under differing environmental conditions, appropriate for the range of environments being regulated. The research must be objective and focus on the practical ecology of stream environments, their adjacent forests, and the food supply for fish. This research will determine where preventive actions (e.g., buffers providing shade) will avoid harm and where management that increases exposure (e.g., dappled cover) is beneficial. In major management zones or areas defined by regulators, fisheries biologists need to identify test streams where fish are present and can be observed before and after treatment. These streams could be tested with potential riparian management options.

The purpose of these tests is to: (a) provide test data capable of defining and meeting safe and productive temperature environments appropriate to climate zones; (b) prescribe suitable forest management along streams, as defined by forest cover and growth, responses of fish, and persistence of downstream temperature changes; and (c) inform regulators as to how forest practice rules might adapt to environmental habitat differences influencing fish.

The proposed testing system provides guidance about where regulation is needed, and if so: (a) where stream reaches may need various amounts of functional shade; and (b) how these alternative prescriptions provide for acceptable levels of water temperatures, primary production, and sources of large woody debris. These would be installed within each of two or three elevation zones in large geographical regions, for example from the Willamette Valley to the Pacific Coast. Perhaps three elevation zones would be sufficient to evaluate how climate affects season-long water temperature patterns for each region. The array of tests might include: (a) complete clear-cuts; (b) <15 m (<50 ft) width, south-side only buffers (as described by Cole and Newton 2013); (c) 22 m (70 ft) width, south-side only buffers; (d) current riparian rule application, both sides; and (e) 50 % greater buffer width than existing requirements, both sides.

Each test stream would include two adjacent reaches about 375 m (1200 ft) long, selected according to presence of fish populations large enough to find representative samples. One of those reaches would not have any harvest, while the reach below would be harvested according to one of the prescriptions described. Each reach would be examined for a 3-year pretreatment period to characterize fish communities, including population, size, and growth rates. The same fish sampling process would be repeated after logging with a time lag suitable for populations to adapt to the changed environment, usually about 3 years. Methods such as those used by Wilzbach et al. (2005) might be needed to isolate reaches to avoid fish movement confusing test results.

In areas where temperature may not be as critical as fish nutrition, a set of treatments may include buffers with mottled cover, such as outlined in the Broadmeadow and Nisbet (2004) description of buffers with various types of openings; their mention of dappled canopy openings and patches of deciduous and coniferous cover leading to seasonal openings for primary production while allowing photosynthesis is creative and objective oriented.

## Discussion and conclusion

Oregon is considering rule changes to the Oregon Forest Practices Act designed to meet the state’s water quality standards, particularly the PCWS. The resource benefits achieved by the rule must be proportional to the harm caused by forest practices (ORS527.714(5)(f)). Wilkerson et al. (2006) described substantially narrower shade buffers in Maine than are being considered for Oregon as adequate to maintain stream temperatures at desirable levels. Fish response data are meager but suggest that some stream openings could be beneficial to fish and that current practices are not negatively affecting fish populations (Mellina and Hinch 2009). Lack of fish data on a range of management alternatives creates a regulatory paradox in that management practices designed to protect water quality and fish habitat could actually diminish both fish productivity and the landowner’s timber values.

A large number of reports reveal that fish food and fish biomass are greater where streams run through clearings than when flowing through unbroken forests (Murphy and Hall 1981; many others). These reports have consistently demonstrated the importance of light on fish-bearing streams as long as direct sunlight is not continuously on water. The prohibition of abusive forest practices (such as equipment operating in the stream or instream wood removal) has been in place for over 30 years. The Oregon Board of Forestry must choose the least burdensome alternative (ORS-527. 714(5) (e)) for resource protection. An early study by Murphy et al. (1981) in the Oregon Cascades found that “…streams traversing open clear-cuts had greater rates of microbial respiration, and greater densities or biomasses of aufwuchs, benthos, drift, salamanders, and trout than did the shaded, forested sites…” This is powerful evidence that maintaining or removing cover can be used as a management tool to protect streams from excessive water temperatures or enhance the fisheries productivity.

We see evidence of a positive fish response to timber harvesting in our own research and the research of others testing the effectiveness of the current Oregon Forest Practices Act (e.g., Newton and Zwieniecki 1996; Newton and Cole 2005; Cole and Newton 2013; Kibler et al. 2013). This evidence suggests that there is no emergency to fish created by the existing OFPA rules, but current rules do minimize management that could favor both forests and fish. Increasing protection from a non-point source activity where water quality changes are minor and diminish rapidly downstream (e.g., Holaday 1992; Zwieniecki and Newton 1999; Johnson 2000) and over time (Summers 1982) is costly, especially when it does not benefit fish. We postulate that to support an abundant fishery, rules must allow positive riparian management to: (a) maintain stream banks and avoid instream wood removal; (b) provide for reasonable amounts of future wood recruitment for stream structure, cover, and allow for associated terrestrial invertebrate production; (c) allow enough light on the water to provide a reasonable level of primary productivity; and (d) provide a favorable range of water temperatures in which moderately well-fed fish are likely to grow near their maximum potential (perhaps 80–90 % of maximum) free of disease. This last element acknowledges the interaction of temperature tolerance and feed abundance outlined by Brett et al. (1982).

Fish are cold blooded. Body temperature and activity vary with water temperature; demand for food varies directly with stream temperature (Brett et al. 1982; Ice et al. 2004); temperatures can be colder as well as warmer than optimum while still supporting an abundant fishery. The range of temperatures fish are exposed to is important. Greene (1950), Brett (1956), and Brett et al. (1982) long ago noted that the interaction of stream temperature and food supply is strong, and that there is a range of several degrees at which fish weight gain varies very little around a healthy rate if fed to satiation; Brett et al. (1982) described how tolerance to rising temperature increases as the season progresses. Greene (1950) was among the first to observe that fish abundance was greater in meadow environments than in a shaded stream despite considerably warmer water. General application of this relationship suggests that fish in very cold water need less extensive buffers than fish in warm water. There is strong evidence that short periods of temperatures above 19 °C (70 °F) are not harmful if foraging is adequate (Ice et al. 2007). The 24 Oregon streams Newton and Zwieniecki (1996), Zwieniecki and Newton (1999), Newton and Cole (2005, 2013), and Cole and Newton (2013, 2015) examined include a range of diurnal changes of 1.2 to 3.1 °C (2.0–5.6 °F). They also observed large temperature variations within 150 m reaches flowing beneath forest cover, representing the influence of highly localized energy sources and sinks. Natural water temperature fluctuations due to streamflow levels, season and time of day, channel exposure, and even disturbance events are much greater than the 0.3 °C (0.5 °F) limit prescribed by the PCWS.

The continuous occupation of existing riparian cover by shrubs or rapidly decaying hardwoods such as red alder (*Alnus rubra*) that reproduce poorly without bare soil will eventually give way to shrub dominance. This fails to provide or maintain a source of durable wood for streams. Current silvicultural options for riparian areas, other than the seldom-used hardwood conversion option, seem to doom the mature conifers identified as a desired future condition. Only by active management will long-term forest management goals be met.

Retention of buffers on the north side may provide for other functions (e.g., favorable relative humidity regimes for amphibians), but not shade. Yet current rules have two equal buffers, one on each side. It may be useful to emphasize the shade-making south side for the best return on the environmental investment if additional shade is needed. Removal of north-side cover may also allow escape of long-wave radiation from water, allowing modest cooling and maintenance of cool streams, as shown by Cole and Newton (2013, see supplement). Local sub-regions may have some storms from directions other than the south, and hence justify trees’ orientation elsewhere for large woody debris recruitment.

Streams experience extreme changes in flow (floods to droughts). Daily and seasonal variations in solar radiation, wind-damage, wildfires, landslides, insect outbreaks, and other disturbances are normal. Clearing of cover and warming of water has had substantial attention, primarily toward negative effects, but fish have survived extreme damage to their environment. Aside from storms, Bisson et al. (2005) reported that following the 1980 eruption of Mt. St. Helens, fish populations thrived in what would otherwise be considered undesirable stream temperatures due to the presence of abundant food supplies. Bisson et al. (2005) observed that previous estimates of fish productivity in a river draining volcanic ash had such high populations of fish that estimation of fish growth was confounded by competition among these super-abundant populations. Heck (2004) found fish growth in a forest watershed after wildfire positively correlated with increased water temperature, presumably owing to increased photosynthesis and aquatic biota. Positive response to wildfire disturbance has been reported elsewhere (Malison and Baxter 2010).

It remains important that rules balance environmental and economic benefits. Ruckelshaus (1989), first US Environmental Protection Agency administrator, noted that “environmental protection and economic development are complementary rather than antagonistic processes…” Forest landowners need to be confident that foregone economic benefits are buying a strong environmental return on investments.

While the focus of riparian forest research has long been on how management affects fish, other beneficial uses have also been studied. Even early research on clearcutting near streams showed that amphibian populations, like fish populations, could respond favorably to increased light and primary production (Murphy et al. 1981). Rundio and Olson (2007) found specific site conditions, such as the presence or absence of downed large wood, and specific species to be important factors affecting salamander response to forest thinning. Dupris and Steventon (1999) found that larval tailed frog densities were lower in streams logged without buffers than in those with them. Bird and bat populations associated with riparian areas may also be affected by changes in forest structure (Skagen et al. 2005). Importantly, forest management is a cycle, so negative impacts to habitat can recover over time as long as populations are not extirpated. Widespread removal of forests due to past harvests and wildfires suggests that these issues can be managed.

It is likely that riparian decisions will be more complex for forest managers if additional site-specific conditions are considered. For riparian areas, most states allow both complete hands-off options and alternative plans where different management strategies can be justified. The State of Washington used a Watershed Analysis approach to set basin-specific forest practice regulations (Montgomery et al. 1995), but this became too costly and cumbersome to persist. Still, the shared experiences from these analyses allowed revisions in the forest practice rules to adopt common management strategies. By “conditioning” forest practices to important site-specific factors, managers will not achieve “perfect” practices for any particular site, but they will more closely balance environmental protection, economic, and ecological needs.

Any proposal that might cause small, temporary increases in water temperature must also address how these changes interact with current and projected global warming. Losses of salmonid habitat are expected from rising water temperatures in the Pacific Northwest (Battin et al. 2007; Ruesch et al. 2012). Water temperature response to climate change is complex, involving air temperatures, streamflow patterns, geology, and other factors (Tague and Grant 2009), but much of the forest stream network will probably remain cool with the influence of groundwater inputs even if snowpacks are diminished. It will be important to maximize productivity in these areas if other habitat sites are compromised. Because water temperature changes rapidly dissipate downstream and riparian forests re-grow to provide shade, significant cumulative effects downstream are unlikely. The fact that salmonids have thrived despite very large temperature changes due to wildfires and volcanic eruptions provides evidence that proposed riparian management practices are unlikely to exacerbate global warming impacts and could actually be used to offset losses in other portions of the stream network. Because stream temperature is a key variable that should be accounted for in setting appropriate riparian management rules, any drift upward in water temperatures due to climate change would also shift the management rules for a particular site.

Habitats in streams or their riparian forests are not currently being considered as factors in deciding management options; warm and cold, high and low elevation, all streams are treated alike. The PCWS seems to imply that any warming from a harvest unit will be harmful and that heat pulses are cumulative. This reasoning ignores natural cooling downstream by heat exchange mechanisms and cold-water mixing (Newton and Zwieniecki 1996; Zwieniecki and Newton 1999) and the benefits of primary productivity resulting from solar radiation. This standard for change in water temperature is substantially smaller than year to year differences in peak temperatures in given locations (Cole and Newton 2013) and the acceptable variance in temperature reported by Brett et al. (1982). The PCWS does not adapt to changing forest conditions or the potential benefits from occasional disturbances events, including timber harvests. It is an anti-degradation standard that does not take a landscape view of management activity (forestry) when applied across a region where harvest and propagation of forests, naturally or industrially, create disturbance events. These disturbance events are not greatly different from natural fires, and both the forests and their associated streams depend on disturbance to create productive conditions during recovery.

Contemporary forest practices have greatly reduced immediate negative impacts, including large water temperature increases observed as a result of historic timber harvesting and management activities. Consideration of how to provide for both productive forests and fisheries is part of both harvesting of timber and fisheries management. There is strong evidence that openings and disturbance in riparian areas can boost cold-water fish production in forest streams. Considering the site-specific conditions of forest reaches, some riparian management, such as creating canopy gaps for enhancement of primary production in cold streams, should be allowed to provide for increased fish food production, and to achieve the long-term silvicultural goals for riparian corridors.
